# Continuous cropping of potato changed the metabolic pathway of root exudates to drive rhizosphere microflora

**DOI:** 10.3389/fmicb.2023.1318586

**Published:** 2024-01-05

**Authors:** Yanhong Xing, Pingliang Zhang, Wenming Zhang, Chenxu Yu, Zhuzhu Luo

**Affiliations:** ^1^College of Resources and Environmental Sciences, Gansu Agricultural University, Lanzhou, China; ^2^Dryland Agriculture Institute, Gansu Academy of Agricultural Sciences, Lanzhou, China; ^3^Department of Agriculture and Biosystem Engineering, Iowa State University, Ames, IA, United States

**Keywords:** potato, continuous cropping obstacle, metabolomics, bacterial community, fungal community

## Abstract

For potato production, continuous cropping (CC) could lead to autotoxicity buildup and microflora imbalance in the field soil, which may result in failure of crops and reduction in yield. In this study, non-targeted metabolomics (via liquid chromatography with tandem mass spectrometry (LC–MS/MS)) combined with metagenomic profiling (via high-throughput amplicon sequencing) were used to evaluate correlations between metabolomics of potato root exudates and communities of bacteria and fungi around potato plants to illustrate the impacts of CC. Potato plants were grown in soil collected from fields with various CC years (0, 1, 4, and 7 years). Metabolomic analysis showed that the contents and types of potential autotoxins in potato root exudates increased significantly in CC4 and CC7 plants (i.e., grown in soils with 4 and 7 years of CC). The differentially expressed metabolites were mainly produced via alpha-linolenic acid metabolism in plant groups CC0 and CC1 (i.e., no CC or 1 year CC). The metabolomics of the groups CC4 and CC7 became dominated by styrene degradation, biosynthesis of siderophore group non-ribosomal peptides, phenylpropanoid biosynthesis, and biosynthesis of various plant secondary metabolites. Continuous cropping beyond 4 years significantly changed the bacterial and fungal communities in the soil around the potato crops, with significant reduction of beneficial bacteria and accumulation of harmful fungi. Correlations between DEMs and microflora biomarkers were established with strong significances. These results suggested that continuous cropping of potato crops changed their metabolism as reflected in the plant root exudates and drove rhizosphere microflora to directions less favorable to plant growth, and it needs to be well managed to assure potato yield.

## 1 Introduction

Potato (*Solanum tuberosum* L.) is an important commodity crop globally as food, feed, and industrial raw material due to its high-yield, strong tolerance against hash environment, and excellent adaptability (Camire et al., 2009). Potato is a good source of proteins, amino acids, minerals, vitamins, and dietary fiber (Camire et al., 2009). Worldwide, acreage of potato planting is increasing every year. High demands of potato create strong motivation for continuous cropping (CC), especially in China where the arable land is limited (Qin et al., 2017a). However, studies showed that the yield of potato from CC fields decreased gradually, by as much as 27, 75, and 85% in years 2, 3, and 4, respectively (Qin et al., 2017a). Such drop in yield calls into question the benefit of continuous cropping for potato.

The deterioration of crop yield during CC is defined as continuous cropping obstacle (CCO), which is most likely resulted from changes in root exudates and soil microflora (Gao et al., 2019, 2021; Han et al., 2022). Although most of root exuded metabolites have beneficial effects to the plant, for example, organic acids are known to increase nutrient mobilization (thus absorption by the roots; Khademi et al., 2010), sugars and amino acids feed the microbiota that could play beneficial roles in plant growth (Kang et al., 2015; Topalovic et al., 2020), and as CC continues, root exudates could increase allelopathic autotoxicity that are detrimental to plant growth and recruit harmful microbes to the plants (Han et al., 2022). Accumulation of autotoxic allelochemicals in soil as a mechanism of CCO has been identified in many plants (Qin et al., 2017b; Wu et al., 2018; Huang et al., 2020), which could affect the health of the plants, and the complex interactions between plants and soil microbes (Gao et al., 2019, 2021). Changes in types and amounts of allelochemicals secreted by plant roots can have impact on the growth and reproduction of soil microbes and change the ratio of pathogenic to beneficial microorganisms in the soil, leading to soil microflora imbalance (Xia et al., 2015; Bonanomi et al., 2016; Jin et al., 2019). Plant autotoxicity was also reported to cause CCO (Xiang et al., 2022). For instance, autotoxicity has been reported to cause replant failure in the continuous cropping of *Angelica sinensis* (Xin et al., 2019), *Lilium davidii* var. *unicolor* (Wu Z. J. et al., 2015), *Nicotiana tabacum* L. (Deng et al., 2017), *Panax quinquefolium* (He et al., 2009), and *Panax notoginseng* (Yang et al., 2015). Many compounds from different root exudates were shown to be autotoxins. Imperatorin, α-spinasterol, vanillin, dibutyl phthalate, and ferulic acid were discovered to be potential autotoxic allelochemicals in *Angelica sinensis* (Xin et al., 2019); the accumulation of phthalic acid was reported to be one of the main CCO factors for *Lilium davidii* var. *unicolor* replantation (Wu Z. J. et al., 2015); dibutyl phthalate, diisobutyl phthalate, and diisooctyl phthalate in root exudates were shown to play important roles in autotoxicity of *Nicotiana tabacum* L. (Deng et al., 2017); some phenolic acids in root exudate of *Panax quinquefolium* and rhizosphere soil have been identified as potential allelochemicals (He et al., 2009); ferulic acid and saponin were shown to inhibit the growth of *P. notoginseng* (Yang et al., 2015). Studies on potato CCO revealed that high concentrations of allelopathic substances secreted by potato roots could produce allelopathy effects on the plants (Soltys-Kalina et al., 2019; Xin et al., 2022; Szajko et al., 2023), and soil microflora around the roots were changed in response to potato CC (Qin et al., 2017a). However, key questions remain to be answered: What are the main autotoxins secreted by potato roots under continuous cropping? Are they changing over time, and how? What are the relationships between soil microflora changes and allelochemical accumulation around potato plants? Answers to these questions are needed for better understanding CCO for potato and for finding ways to overcome the CCO to promote potato production.

This study aimed to understand how potato CC can alter root exudates and induce changes in rhizosphere microorganisms. Potato plants were grown in pot groups with soil collected from fields with different continuous cropping years (0, 1, 4, and 7, namely, CC0, CC1, CC4, and CC7 groups). Rhizosphere and bulk soil from each pot were collected and analyzed to assess changes of bacterial and fungal communities around the plants. Meanwhile, root exudates from the plants were collected and analyzed for changes and metabolic pathways by non-targeted metabolomics. By identifying biomarkers of root exudates and microbes (i.e., rhizosphere and bulk soil) in samples from different CC groups, the relationships between microbes and root exudates were established to clarify the chemotaxis or avoidance tendencies of microbes toward the root exudates and reveal the mechanisms of CCO for potato.

## 2 Materials and methods

### 2.1 Pot experiment design

Soil for the potato pot experiments was collected in 2021 from the positioning testing site located in the National Soil Quality Stability Observation and Experiment Station of Gansu Academy of Agricultural Sciences, Gansu Province, North-west China (104 ° 36′E, 35 ° 35ʹN). The average altitude of the site is 1970 m, the average annual temperature is 6.2°C, the average annual precipitation is 415 mm, the average annual evaporation is 1,531 mm, and the frost-free period is 146–149 day/year. The physical and chemical properties of the soil in 2015 were organic carbon 10.62 g·kg^−1^, total nitrogen 0.78 g·kg^−1^, available phosphorus 10.12 mg·kg^−1^, available potassium 163.8 mg·kg^−1^, and pH 8.32. The characteristics of the soil changed over 7 years of CC, as shown in Table 1.

**Table 1:** Soil physical and chemical properties under different treatments (2021).

Factors	CCO	CC1	CC4	CC7
Total nitrogen (g·kg^−1^)	0.80 ± 0.02b	0.76 ± 0.01b	0.78 ± 0.02b	1.33 ± 0.09a
Total phosphorus (g·kg^−1^)	0.88 ± 0.01a	0.68 ± 0.01c	0.67 ± 0.003c	0.70 ± 0.01b
Available phosphorus (mg·kg^−1^)	15.52 ± 0.02a	5.37 ± 0.02d	7.87 ± 0.02c	14.31 ± 0.02b
Available potassium (mg·kg^−1^)	81.63 ± 0.18d	98.83 ± 0.07c	128.57 ± 0.12b	168.50 ± 0.21a
pH	8.22 ± 0.01d	8.59 ± 0.03b	8.71 ± 0.01a	8.35 ± 0.02c

Values are mean ± SE in triplicate replicates. Different lowercase letters indicate significant differences with a *p* < 0.05 based on the analysis of variance.

Topsoil (0–20 cm) from fields of rotation (maize/potato, with maize planted the previous season) and continuous cropping of 1, 4, and 7 years (potato alone) were collected in 2021 and sieved through a 10.0 mm sieve to remove large root chips and stones after air-drying, four groups were set up and denoted by CC0 (rotation control), CC1 (continuous cropping for 1 year), CC4 (continuous cropping for 4 years), and CC7 (continuous cropping for 7 years), and each group included six pots for replication. Each pot had 2.0 kɡ air-dried soil, with fertilizers applied at 0.1 gN/kg soil, N/P = 2:1 (urea for N, and potassium dihydrogen phosphate for P). In each pot, two plants (Longshu no.3) were seeded at 9 cm apart and 4 cm from the surface. Pots were placed in the greenhouse on campus of Gansu Agricultural University from 10 May to 14 July of 2021.

### 2.2 Sample collection

Potato plants in the pot were taken out as a whole at the blossoming stage, 65 days after seeding, and paired rhizosphere soils (R) and bulk soils (B) were collected with the “soil adhering to fine roots after shaking” method (Huo et al., 2022); in brief, soil was first collected by shaking the roots gently till dropping of large soil aggregates ceased, and the soil was defined as bulk soil (BS); then, soil adhering to the fine roots was collected by brushing the roots with sterile brushes, which was defined as rhizosphere soil (RS). Soil samples were then split into two parts: One part was air-dried, and another was frozen at −80°C. After RS collection, the plants were put into a 250 mL conical flask, and ultrapure water was added until the roots were completely submerged. After 24 h (16 h under light and 8 h in the dark), the solution was collected and centrifuged at 1200 rpm, at 4°C for 15 min; then, the supernatant was filtrated by a 0.22 μm microfilter, and the filtrate was freeze-dried to get the root exudates, which was frozen-stored at −80°C. The root exudates alongside with soil samples from three pots (randomly selected) of each group were sent to Biomarker Technologies Co., Ltd. (Beijing, China) for metabolomics analysis and gene sequencing.

### 2.3 Non-targeted metabolomic analysis

Untargeted metabolites in the root exudates were determined using a liquid chromatography with tandem mass spectrometry (LC–MS/MS) platform (Biomarker Technologies Co., Ltd.). In brief,1 mL extraction liquid (methanol: acetonitrile: water = 2:2:1) was added into 50 mg of the samples and vortexed for 30 s, and steel balls were added and ground in a 45 Hz grinder for 10 min and ultrasonicated in an ice water bath for 10 min (Dunn et al., 2011). The sample was then sat at 20°C for 1 h to precipitate the proteins and then centrifugated at 4°C, 12000 rpm for 15 min. 500 μL of supernatant were then moved into an EP tube and dried in a vacuum concentrator; then, 160 μL of an extraction liquid (acetonitrile: water = 1:1) was added to redissolve the sample. It was then vortexed for 30 s, ultrasonicated for 10 min (in ice water bath), and centrifugated for 15 min (4°C, 12,000 rpm). Finally, 120 μL of the supernatant were used for Ultra-High Performance Liquid Chromatography-Q Exactive (UHPLC-QE) orbital trap/mass spectrometry analysis. The LC/MS system for metabolomics analysis is composed of Waters Acquity I-Class PLUS ultra-high performance liquid tandem Waters Xevo G2-XS QT of high-resolution mass spectrometer, and the column used was from Waters Acquity UPLC HSS T3 column (Waters Corp., Milford, CT, United States).

### 2.4 DNA extraction and sequencing

The total genomic DNA from 0.5 g of both BS and RS samples of each group was extracted with the TGuide S96 Magnetic Soil/Stool DNA Kit (Tiangen Biotech (Beijing) Co., Ltd.) according to manufacturer instructions, and the DNA concentration of the samples was measured with the Qubit dsDNA HS Assay Kit and Qubit 4.0 Fluorometer (Invitrogen, Thermo Fisher Scientific, Oregon, United States). PCR amplification was performed for each soil DNA extract in triplicate and combined into a single composite sample. Fungal ITS region was amplified using the primer pair ITS1F (5’-CTTGGTCATTTAGAGGAAGTAA-3′) and ITS2 (5’-GCTGCGTTCTTCATCGATGC-3′) (Yang et al., 2017), and the thermal cycling conditions were 95°C for 5 min, 15 cycles of 95°C for 1 min, 50°C for 1 min, and 72°C for 1 min, followed by 72°C for 7 min. Bacterial 16S rRNA gene V3-V4 region of 16S rRNA was amplified using the primer pair 338F (5’-ACTCCTACGGGAGGCAGCA-3′) and 806R (5’-GGACTACHVGGGTWTCTAAT-3′) (Wang et al., 2020), and the thermal cycling conditions were pre-denaturation at 98°C for 2 min, denaturation at 98°C for 15 s, annealing at 55°C for 30 s, extension at 72°C for 30 s, and final extension at 72°C for 5 min (30 cycles). The PCR amplicons were gel-purified with Agencourt AMPure XP Beads (Beckman Coulter, Indianapolis, IN). The resultant PCR products were combined at equimolar concentrations and use Illumina NovaSeq 6000 (Illumina, Santiago CA, United States) for sequencing (250 × 250 bp) in Beijing Biomarker Technologies Co., Ltd. (Beijing, China).

Paired-end (PE) reads obtained from previous steps were assembled by USEARCH (version 10) (Segata et al., 2011) and followed by chimera removal using UCHIME (version 8.1) (Quast et al., 2013). The high-quality reads generated by the above steps will be used for subsequent analysis.

### 2.5 Data processing and analysis

The MS raw data were collected using MassLynx software (version 4.2, Waters Corp., Milford, CT, United States) and processed by the Progenesis QI software (Waters Corp., Milford, CT, United States). The METLIN database (Waters Corp., Milford, CT, United States) and an in-house database (Biomarker Technologies Co., LTD.) were used for peak annotation and identification of various compounds (Zhang et al., 2022). The projection (VIP > 1), Student’s *t*-test (*p* < 0.05), and |log_2_FC| ≥ 0.58 were used as criteria to screen for differentially expressed metabolites (DEMs), and the threshold (VIP > 1, *p* < 0.05, |log2FC| ≥2.32) was used to screen the DEMS that varied greatly, defined as greatly differentially expressed metabolites (GDEMs).

The orthogonal partial least-squares discrimination analysis (OPLS-DA; R, 3.3.2, ropls packages) and the principal component analysis (PCA; R, 3.1.1, “scales,” “ggplot2,” “ggrepel,” “scatterplot3d” packages) were used to distinguish different groups of overall differences in metabolic profile, the volcano map (R, 3.1.1, “ggplot2” packages) was used for visualization of the differential substance, and the KEGG pathway of the DEMs was mapped in R (version 3.1.1, “clusterprofiler,” “enrichplot” packages; Wu et al., 2022).

Operational taxonomic units (OTUs) were clustered with 97% similarity using Usearch (version 10.0) software. Beta diversity analysis of samples was evaluated by QIIME2 software (version 2020.6), and non-metric multidimensional scaling (NMDS) analysis of Gower distance and sperman-approx distance was used to show the divergence of the rhizosphere microbial communities for bacterial and fungal, respectively. NMDS analysis of Hellinger distance and binary-chord distance was used to show the divergence of bacterial and fungal communities in both rhizosphere and bulk soil, respectively. The linear discriminant analysis (LDA) effect size (LEfSe) was used to identify biomarker with statistical difference using R (version3.1.1, stats package) and python (version1.0.0, scipy package; Segata et al., 2011). Correlation network analysis was used to identify key species using Cytoscape (version 3.7.1) and R (version3.1.1, igraph package; Shannon et al., 2003; Zheng et al., 2018), genera with average relative abundances higher than 0.1% were subject to Spearman’s correlation analysis, and bacterial and fungal genera with Spearman’s correlation >0.8 or < −0.8 and significance *p* < 0.01 were used to establish microbial networks.

The related network between metabolites and genus was established with Pearson’s correlation coefficient (PCC) > |0.8| and a *p* < 0.05 and visualized with Cytoscape (version 3.7.1; Pyo et al., 2022). The high-level correlation network analysis (Spearman’s correlation (*r* > 0.8 and *p* < 0.05)) between top 20 microbe and DEMs was involved in metabolic pathways, the correlation heatmap between DEMs, and the significantly differentiated microbes from rhizosphere and bulk soil with *t*-test (*p* < 0.05).

## 3 Results

### 3.1 Physiological indicators of plants grown in soils with different CC years

Dry weight, stem thickness, and height of plants all decreased in groups of increasing CC years (Table 2), while the amount of root secretion increased significantly, indicating CC depressed the potato growth, while promoted root secretion, which consumed large amount of photosynthates. Biomass of the plant (i.e., dry weight) and physiological indicators (stem thickness and height) suggested that plants grown with soil under the growing impact of CCO were getting less healthy. Meanwhile, the plants were producing more root secretion, especially in soil with longer CC, which were signs of plants responding to the changing soil environment. These signs were further clarified by the metabolomic analysis of the root exudates.

**Table 2:** Plant physiological indicators under different CC years.

Factors	CC0	CC1	CC4	CC7
Dry weight (g.pot^−1^)	3.79 ± 0.09a	2.68 ± 0.06b	2.69 ± 0.06b	2.54 ± 0.09b
Stem thick (mm)	5.55 ± 0.15a	5.39 ± 0.09ab	4.91 ± 0.26b	4.88 ± 0.22b
Plant height (cm)	25.95 ± 0.45a	25.63 ± 0.39a	25.62 ± 0.17a	25.37 ± 0.20a
Mass of root secretion (g.plant^−1^)	0.0408 ± 0.01b	0.0469 ± 0.01ab	0.0706 ± 0.01ab	0.0728 ± 0.01a

Values are mean ± SE in triplicate replicates. Different lowercase letters indicate significant differences with a *p* < 0.05 based on the analysis of variance.

### 3.2 Metabolomic analysis of root exudates of potato with CCO

**Figure 1:**
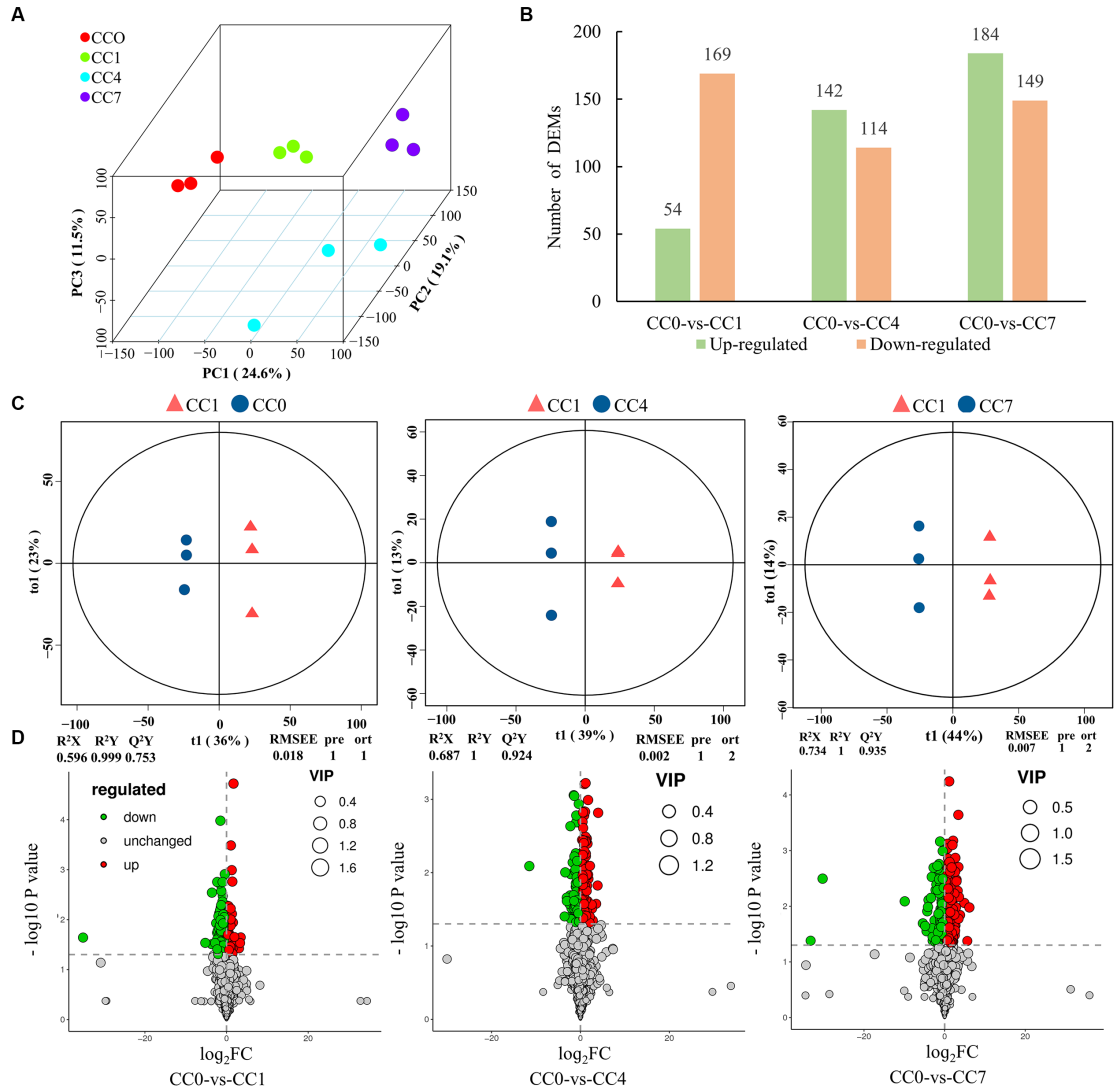
Metabolomic analysis of root exudates. **(A)** Principal component analysis (PCA) of root exudates, where the x-axis represents the first principal component, the Y-axis represents the second principal component, and the Z-axis represents the third principal component. **(B)** Number of upregulated and downregulated differentially expressed metabolites (DEMs). **(C)** Orthogonal partial least-squares discrimination analysis (OPLS-DA) scores plot of metabolites in soil between control and treatments, the parameters of the model, including R^2^X, R^2^Y, Q^2^Y, RMSEE (root mean squared error), pre (predict the number of components), and ort (the number of orthogonal components), where R^2^X and R^2^Y denote the percentage of X and Y matrix information that can be interpreted by the OPLS-DA classification model, respectively, and Q^2^Y is calculated by cross-validation to evaluate the predictive power of the OPLS-DA model, and the closer these metrics are to 1, the better the OPLS-DA model fits the data. **(D)** Volcano map of differential metabolites, green dots represent downregulated metabolites, red dots represent upregulated metabolites, and gray dots represent no-differential metabolites.

A total of 27,975 peaks were detected in 12 samples by metabolome characterization and quantitative analysis, of which 1,722 metabolites were annotated (Supplementary Table S1), and these compounds mainly included fatty acyls, prenol lipids, carboxylic acids and derivatives, and organooxygen compounds (Supplementary Figure S1). PCA analysis and OPLS-DA model showed that metabolite data from different groups (CC0, CC1, CC4, and CC7) formed clusters that are completely separated (Figures 1A,C), indicating that the metabolites from root exudates of potato grown in pots with soil from different CC fields (0, 1, 4, and 7 years, respectively) were significantly different. Analysis of the root exudates showed that CC significantly changed the chemical composition of the root exudates, and longer the continuous cropping years, greater the differences shown in the differentially expressed metabolites (DEMs). Upregulated DEMs in CC1, CC4, and CC7 were 54, 142, and 184, and downregulated DEMs were 169, 114, and 149 (Figures 1B,D), respectively, and the entire lists are given in Supplementary Tables S2–S4. It is worth noting that more upregulated DEMs are produced with the increase of CC years.

Notably the upregulated DEMs are autotoxins reported for various plants. Eight potential autotoxins including phenols, flavonoids, coumarins, and alkaloids were found in group CC1, 18 potential autotoxins including phenols, flavonoids, coumarins, alkaloids, and terpenoids were found in group CC4, and 30 potential autotoxins including phenols, coumarins, flavonoids, alkaloids, terpenoids, and fatty acids were found in group CC7 (Supplementary Table S5). Both the types and the quantities of phenolic compounds increased significantly with CC years; in addition, the GDEMs (Supplementary Table S6) may play important roles in CCO for potato, for example, 1-(4-hydroxyphenyl) ethanol (phenols) in CC4, and 1,2-naphthoquinone, 4-methylumbelliferone (coumarins), 1-(4-hydroxyphenyl) ethanol, phloretin (flavonoids), and psoralen (coumarins) in CC7 appeared to be the key autotoxins that could negatively impact the potato yields in these groups.

### 3.3 Enrichment of differentially expressed metabolites as affected by CC

**Figure 2:**
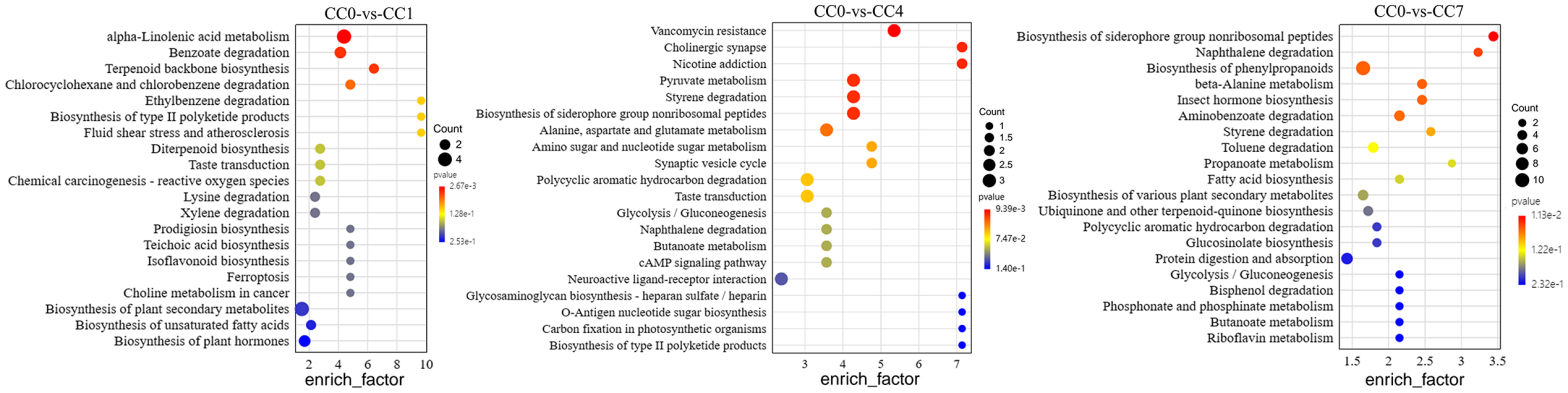
KEGG map of metabolic pathways with significant enrichment of DEMs between groups CC0 and CC1, CC4, and CC7. The x-axis is the enrich factor of the DEMs enriched in the pathway, and the y-axis lists the names of the pathways; the color depth of the dot represents the *p*-value, the redder the color, the more significant the enrichment, and the size of the dot represents the number of DMEs enriched.

Analysis of metabolic pathways can reveal the metabolic process leading to the production of the DEMs among groups. KEGG map of metabolic pathways (Figure 2) showed that DEMs were mainly enriched via alpha-linolenic acid metabolism, biosynthesis of plant secondary metabolites, benzoate degradation, and biosynthesis of plant hormones in group CC1; pyruvate metabolism, styrene degradation, biosynthesis of siderophore group non-ribosomal peptides, alanine, aspartate, and glutamate metabolism in group CC4; phenylpropanoid biosynthesis, protein digestion and absorption, aminobenzoate degradation, toluene degradation, and biosynthesis of various plant secondary metabolites in group CC7, respectively.

**Figure 3:**
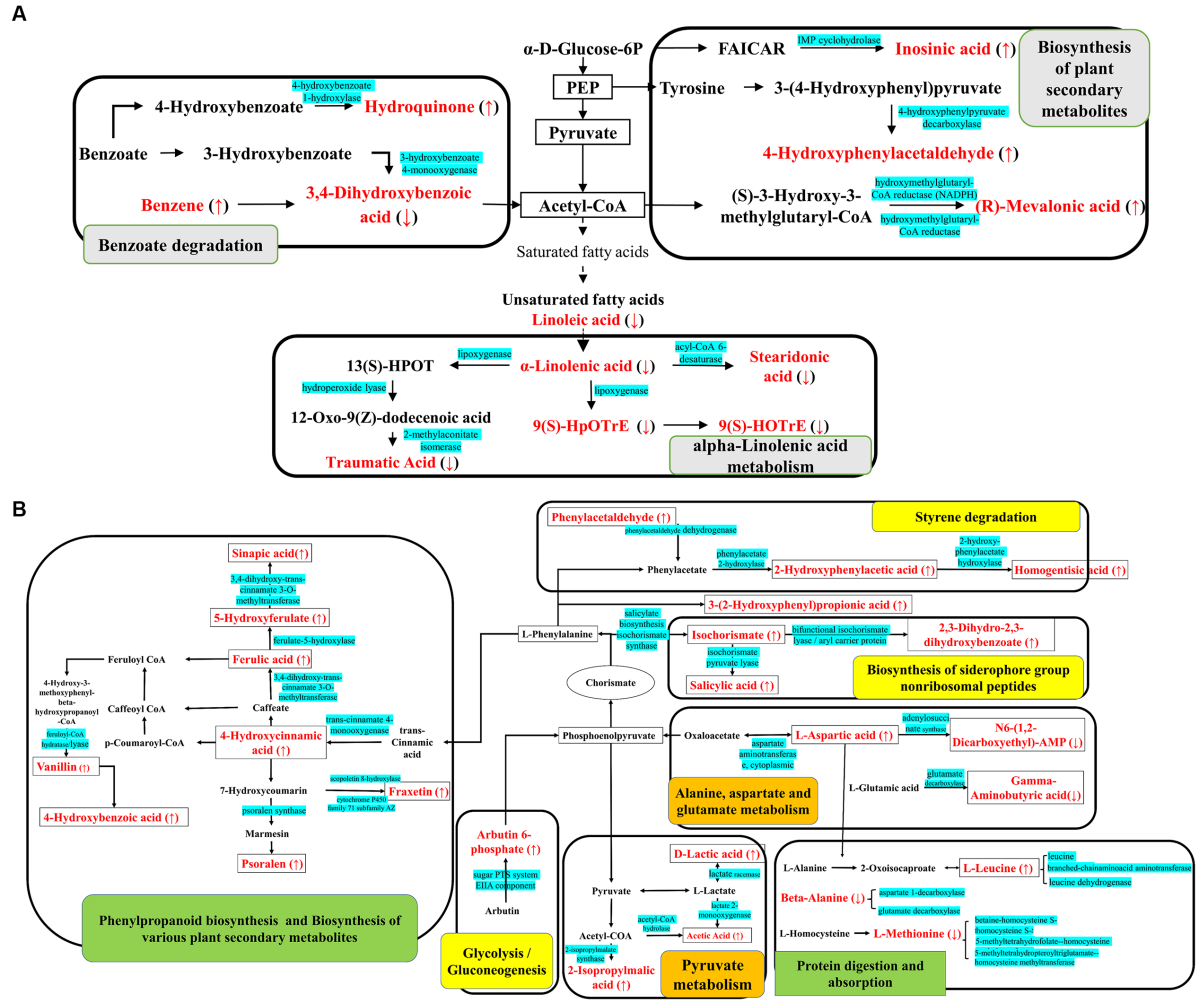
KEGG metabolic pathway map of **(A)** CC1 and **(B)** CC4 and CC7. The red marked are the DEMs, with ↑ indicating upregulated and ↓ indicating downregulated, and the blue marked are the enzymes involved in the synthesis of the metabolites. Gray shows the specific metabolic pathway in CC1, green shows the specific metabolic pathway in CC7, orange shows the specific metabolic pathway in CC4, and yellow shows the common metabolic pathways in both CC4 and CC7.

Notably, as shown in Figure 3, biosynthesis of siderophore group non-ribosomal peptides, styrene degradation, butanoate metabolism, glycolysis/gluconeogenesis, naphthalene degradation, and polycyclic aromatic hydrocarbon degradation was all boosted in groups CC4 and CC7. Further analysis showed that alpha-linolenic acid pathway was downregulated in group CC1, while phenolic acids produced from L-phenylalanine were upregulated in groups CC4 and CC7. This indicated that the metabolic pathways of CC1 plants were different from that of CC4 and CC7 plants, and the metabolic pathways of CC4 and CC7 plants were dominated by the synthesis of autotoxins (e.g., phenols and coumarins).

### 3.4 Analysis of rhizosphere microbial communities in different CC groups

**Figure 4:**
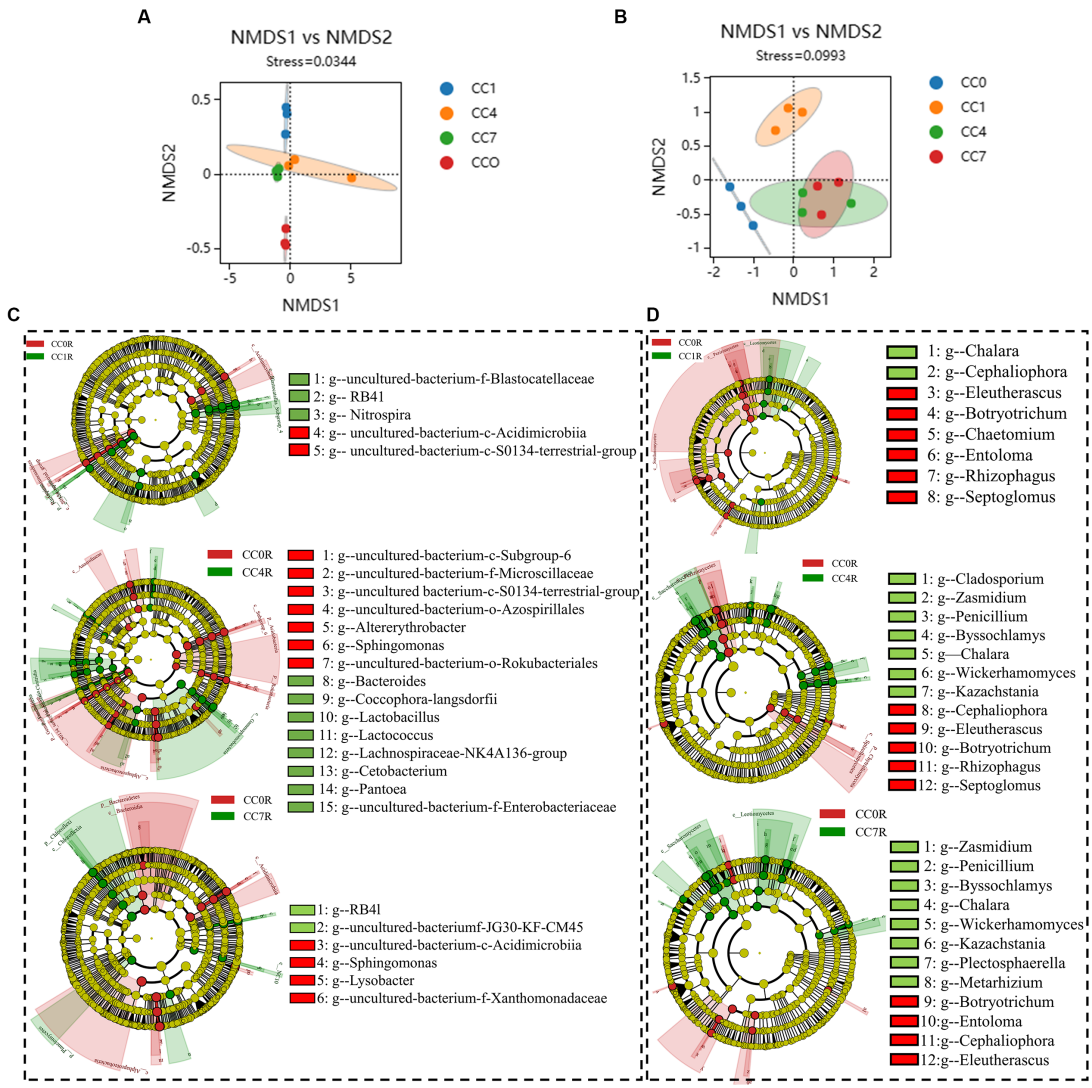
Differential analysis of soil microbial communities in group CC. **(A)** Bacterial non-metric multidimensional scaling (NMDS). **(B)** Fungal NMDS, each dot in the graph represents a sample, different colors represent different groups, and the ellipse circle represents its 95% confidence ellipse. **(C)** Bacterial linear discriminant analysis (LDA) effect size (LEfSe) analysis. **(D)** Fungal LEfSe analysis. The figures show the genera with LDA score greater than 3.5.

As shown in Figures 4A,B NMDS analyses of rhizosphere bacteria and fungi showed that groups CC4 and CC7 clustered closely together, and clearly separated from groups CC0 and CC1, indicating continuous cropping significantly changed the microbial community structure in the RS soil, especially as the CC years grew.

Bacterial LEfSe analysis (Figure 4C) showed that *RB41*, *Nitrospira,* and *uncultured-bacterium-f-Blastocatellaceae* were significantly elevated, while *uncultured-bacterium-c-S0134-terrestrial-group* and *uncultured-bacterium-c-Acidimicrobiia* were significantly decreased in group CC1. *Bacteroides, Lactobacillus, uncultured-bacterium-f-Enterobacteriaceae, Lactococcus, Coccophora langsdorfii, Cetobacterium, Lachnospiraceae-NK4A136-group, and Pantoea* were significantly elevated while *uncultured-bacterium-c-Subgroup-6*, *Sphingomonas*, *uncultured-bacterium-f-Microscillaceae*, *uncultured-bacterium-c-S0134-terrestrial-group*, *Altererythrobacter*, *uncultured-bacterium-o-Rokubacteriales,* and *uncultured-bacterium-o-Azospirillales* were significantly decreased in group CC4, *uncultured-bacterium-c-JG30-KF-CM45* and *RB41* were significantly elevated while *Sphingomonas*, u*ncultured-bacterium-f-Xanthomonadaceae*, *uncultured-bacterium-c-Acidimicrobiia,* and *Lysobacter* were significantly decreased in group CC7.

Fungal LEfSe analysis (Figure 4D) showed that *Cladosporium* in group CC4, *Plectosphaerella* and *Metarhizium* in group CC7, *Kazachstania*, *Byssochlamys*, *Wickerhamomyces*, *Penicillium,* and *Zasmidium* in both groups CC4 and CC7, *Chalara* in groups CC1, CC4, and CC7 were all significantly elevated after continuous cropping, while *Chaetomium* in group CC1, *Entoloma*, *Rhizophagus* and *Septoglomus* in groups CC4 and CC7, *Cephaliophora*, *Botryotrichum* and *Eleutherascus* in groups CC1, CC4, and CC7 were decreased significantly after continuous cropping.

**Figure 5:**
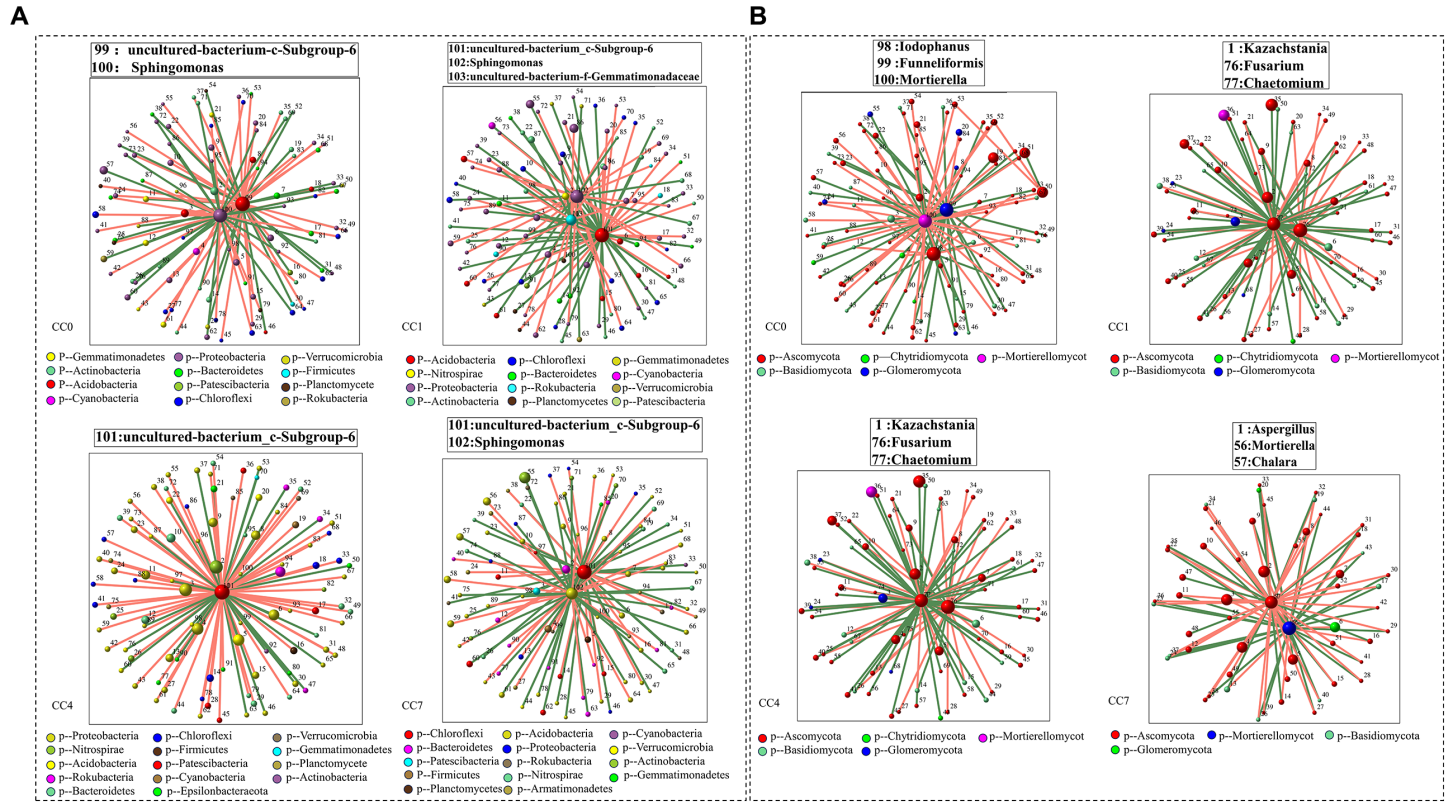
Correlation network diagram of each group [**(A)** bacteria; **(B)** fungi]. Red lines represent significant positive relationships (Spearman’s correlation, *r* > 0.8 and *p* < 0.01), and green lines denote negative relationships (Spearman’s correlation, *r* < 0.8 and *p* < 0.01). The genera shown on these figures are the module hub in each network diagram (ZI > 2.5 and PI < 0.62).

Analysis of microbial correlation networks was conducted to reveal the key microbial species (e.g., bacteria and fungi) under different continuous cropping conditions. As shown in Figure 5, *Uncultured-bacterium-c-Subgroup-6* was identified to be a key bacterium in all CC groups, *Sphingomonas* was a key bacterium in groups CC0, CC1, and CC7, while its relative abundance reduced to minimum in group CC4. Key fungal species under different continuous cropping conditions were *Iodophanus*, *Funneliformis,* and *Mortierella* in group CC1, *Aspergillus*, *Mortierella,* and *Chalara* in groups CC1 and CC7, *Kazachstania*, *Fusarium* and *Chaetomium* in group CC4, respectively. Notably, CC4 showed different characteristics comparing to CC1 and CC7. It should also be noted that the relative abundance of *Chalara* and *Aspergillus* in groups CC4 and CC7 was all significantly higher than those in group CC0, while the opposite was observed for *Mortierella,* whereas continuous cropping seemed to reduce its relative abundance, which went down from that of CC0, the relative abundance of microorganisms was given in Supplementary Table S7.

### 3.5 Analysis of rhizosphere via bulk soil microbiota

**Figure 6:**
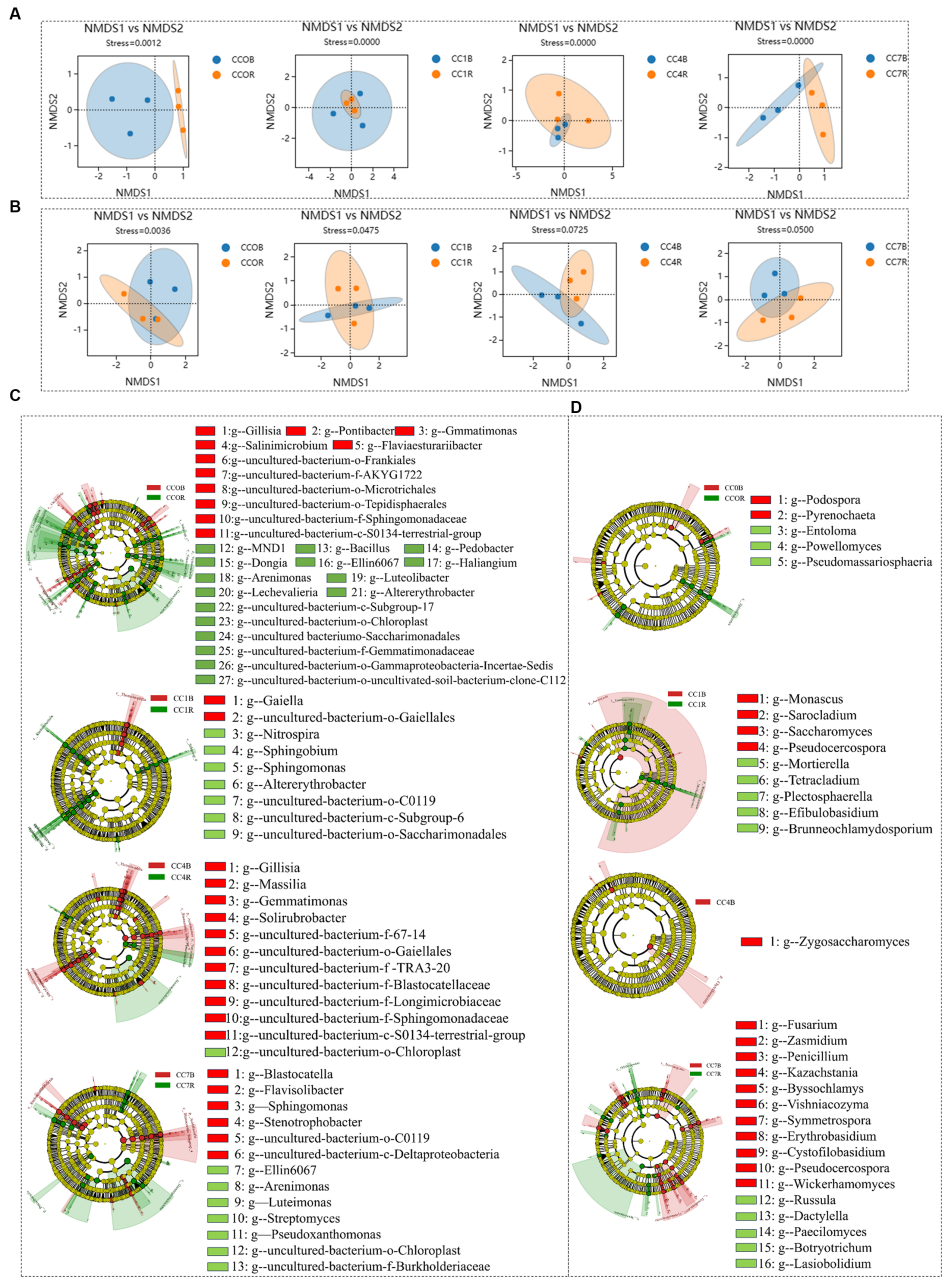
Differential analysis between rhizosphere and bulk soil microbiota in each group. **(A)** Bacterial non-metric multidimensional scaling (NMDS). **(B)** fungal NMDS, each dot in the graph represents a sample, different colors represent different groups, and the ellipse circle represents its 95% confidence. **(C)** Bacterial linear discriminant analysis (LDA) effect size (LEfSe) analysis. **(D)** Fungal LEfSe analysis, the genera with LDA score greater than 3.0.

As shown in Figures 6A,B NMDS analyses showed that the rhizosphere and bulk soil microbial communities of bacteria and fungi in each group were separated clearly, indicating that root exudates significantly influenced the formation of unique rhizosphere microbiota different from that of the bulk soil.

Bacterial LEfSe analysis between rhizosphere and bulk soil of different CC groups (Figure 6C) showed that for CC0, *uncultured-bacterium-f-Gemmatimonadaceae, uncultured-bacterium-o-Saccharimonadales, uncultured-bacterium-o-Gammaproteobacteria-Incertae-Sedis, uncultured-bacterium-c-Subgroup-17, uncultured-bacterium-o-Chloroplast, uncultured-bacterium-o-uncultivated-soil-bacterium-clone-C112, Lechevalieria, Pedobacter, Bacillus, Dongia, Altererythrobacter, Sandaracinobacter, Haliangium, Ellin6067, MND1, Arenimonas, and Luteolibacter* were predominant in the rhizosphere microbiota, while *uncultured-bacterium-f-Gemmatimonadaceae, uncultured-bacterium-o-Saccharimonadales, uncultured-bacterium-o-Gammaproteobacteria-Incertae-Sedis, uncultured-bacterium-c-Subgroup-17, uncultured-bacterium-o-Chloroplast, uncultured-bacterium-o-uncultivated-soil-bacterium-clone-C112, Flaviaesturariibacter, Pontibacter, Gillisia, Salinimicrobium, Gemmatimonas* were predominant in the bulk soil. For CC1, *uncultured-bacterium-o-Saccharimonadales, uncultured-bacterium-c-Subgroup-6, uncultured-bacterium-o-C0119, Sphingobium, Nitrospira, Sphingobium*, and *Altererythrobacter* were predominant in the rhizosphere, while *uncultured-bacterium-o-Gaiellales* and *Gaiella* were predominant in the bulk soil. For CC4, *uncultured-bacterium-o-Chloroplast* was predominant in the rhizosphere, while uncultured-bacterium-f-TRA3-20, *uncultured-bacterium-f-Sphingomonadaceae, uncultured-bacterium-f-67-14, uncultured-bacterium-f-Blastocatellaceae, uncultured-bacterium-o-Gaiellales, uncultured-bacterium-c-S0134-terrestrial-group, Uncultured-bacterium-f-Longimicrobiaceae, Solirubrobacter, Gaiella*, and *Massilia* were predominant in the bulk soil. For CC7, *uncultured-bacterium-o-Chloroplast, Arenimonas, Pseudoxanthomonas, Streptomyces, Ellin6067, Litorilinea*, and *Luteimonas* were predominant in the rhizosphere, while uncultured-bacterium-o-C0119, *uncultured-bacterium-c-Deltaproteobacteria, Stenotrophobacter, Corynebacterium-1, Blastocatella, Flavisolibacter, Sphingomonas* were predominant in the bulk soil.

Fungal LEfSe analysis between rhizosphere and bulk soil of different CC groups (Figure 6D) showed that for CC0, *Powellomyces*, *Pseudomassariosphaeria*, and *Entoloma* were predominant in the rhizosphere, while *Pyrenochaeta* was predominant in the bulk soil; For CC1, *Mortierella*, *Brunneochlamydosporium*, *Tetracladium*, *Efibulobasidium*, and *Plectosphaerella* were predominant in the rhizosphere, while *Monascus*, *Pseudocercospora*, *Sarocladium*, and *Neodidymella* were predominant in the bulk soil; for CC4, *Zygosaccharomyces* was predominant in the bulk soil; for CC7, *Paecilomyces*, *Botryotrichum*, *Russula*, *Lasiobolidium*, and *Dactylella* were predominant in the rhizosphere, while *Symmetrospora*, *Pseudocercospora*, *Phialophora*, *Bipolaris*, *Vishniacozyma*, *Byssochlamys*, *Penicillium*, *Zasmidium*, *Wickerhamomyces*, *Erythrobasidium*, *Fusarium*, and *Kazachstania* were predominant in the bulk soil. As a general observation, it was discovered that in CC1 (1 year of continuous cropping), beneficial bacteria were enriched in the rhizosphere, while in CC7, pathogens and yeasts such as *Fusarium*, *Pseudocercospora*, and *Wickerhamomyces* became predominant in the bulk soil, which could be a significant contributor to the reduction in potato yield.

### 3.6 The relationships between microbiota and DEMs in different CC groups

**Figure 7:**
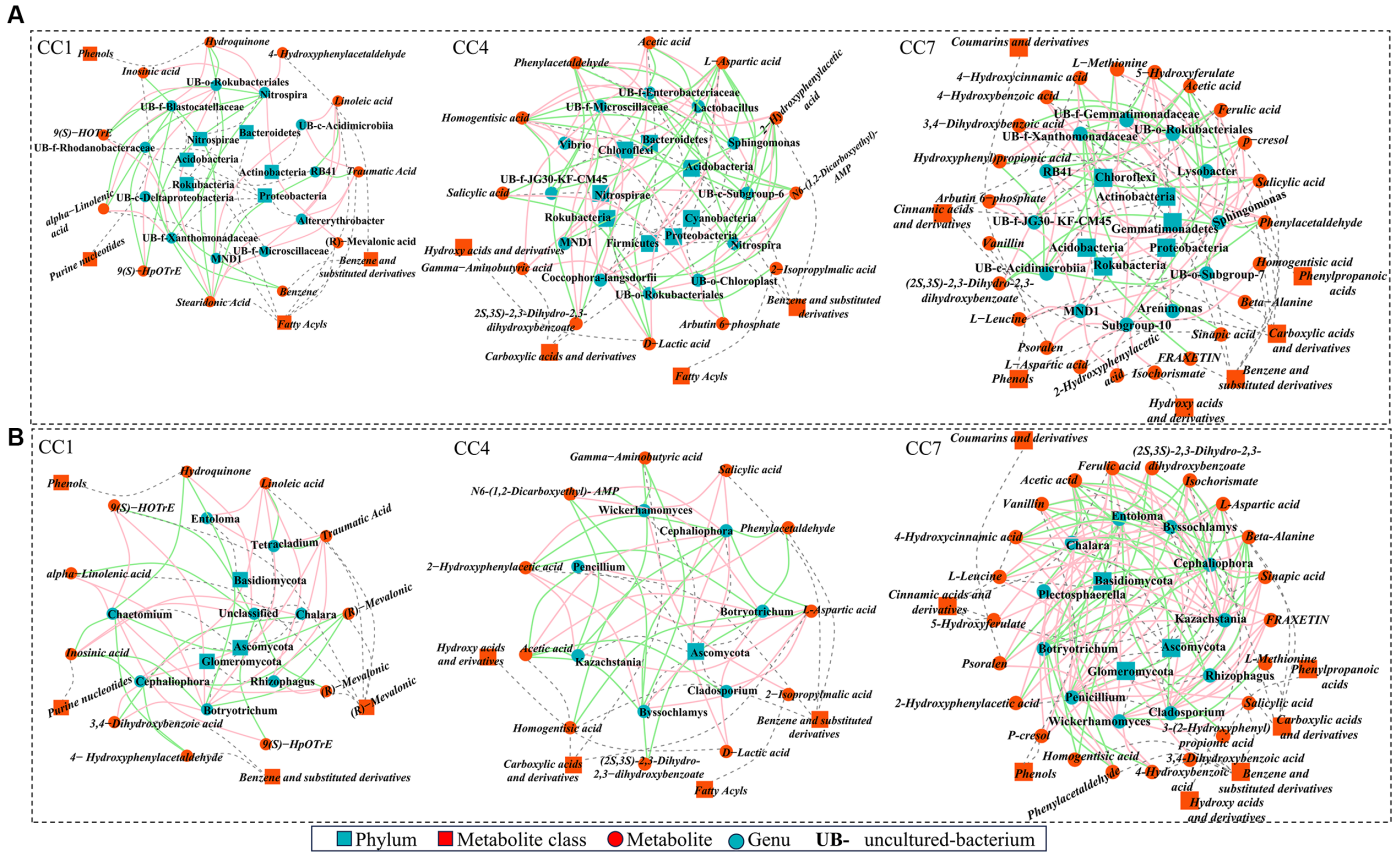
High-level correlation network analysis (Spearman’s correlation, *r* > 0.8 and *p* < 0.05) between top 20 rhizosphere microbes [**(A)** bacteria; **(B)** fungi] and the differentially expressed metabolites (DEMs) involved in metabolic pathways. Red and green lines denote positive and negative relationships, blue squares and dots represent microbe phylum and genus, red squares and dots represent metabolite class and metabolites, and gray dashed line represents affiliation.

Correlation network between top 20 microbes (based on relative abundance) and DEMs involved in metabolic pathways was generated. As shown in Figures 7A,B connections between rhizosphere microflora (both bacteria and fungi) to DEMs could be identified to suggest that the changes of potential autotoxins could be originated from changes of rhizosphere microflora due to continuous cropping.

#### 3.6.1 The relationships between rhizosphere bacterial communities and metabolites

As shown in Figure 7A, in group CC1, key DEMs were produced via the metabolic pathways of α-linolenic acid metabolism, biosynthesis of plant secondary metabolites, and benzoate degradation. These 12 DEMs (e.g., fatty acids, benzene and derivatives, phenols, and purine nucleotides (linoleic acid, α-linolenic acid, (R)-mevalonic acid, traumatic acid, etc.)) were shown to be significantly (*p* ≤ 0.05) or extremely significantly (*p* ≤ 0.01) correlated with 11 bacterial genera. For example, *Nitrospira* was positively correlated with 4-hydroxyphenylacetaldehyde, (R)-mevalonic acid, hydroquinone, and inosinic acid and negatively correlated with 9S-hydroperoxy-10E,12Z,15Z-octadecatrienoic acid (9(S)-HpOTrE), stearidonic acid, 9(S)-hydroxy-10(E),12(Z),15(Z)-octadecatrienoic acid (9(S)-HOTrE), and α-linolenic acid; *RB41* was negatively correlated with traumatic acid, stearidonic acid, and linoleic acid.

In group CC4, metabolic pathways of pyruvate metabolism, alanine, aspartate and glutamate metabolism, biosynthesis of siderophore group non-ribosomal peptides, styrene degradation, and glycolysis/gluconeogenesis were the key processes accounting for 13 DEMs of importance (e.g., benzene and substituted derivatives, carboxylic acids and derivatives, hydroxyl acids and derivatives, and fatty acids [salicylic acid, homogentisic acid, 2-hydroxyphenylacetic acid, L-aspartic acid, etc.]). They were significantly (*p* ≤ 0.05) or extremely significantly (*p* ≤ 0.01) correlated with 12 bacterial genera. Among them, *uncultured-bacterium-c-Subgroup-6* was negatively correlated with arbutin 6-phosphate, salicylic acid, L-aspartic acid, 2-hydroxyphenylacetic acid, and homogentisic acid and positively correlated with gamma-aminobutyric acid, N6-(1,2-dicarboxyethyl)-AMP. *Lactobacillus* and *uncultured-bacterium-f-Enterobacteriaceae* were positively correlated with (2S,3S)-2,3-dihydro-2,3-dihydroxybenzoate, L-aspartic acid, 2-hydroxyphenylacetic acid, phenylacetaldehyde, and homogentisic acid and negatively correlated with N6-(1,2-dicarboxyethyl)-AMP, D-lactic acid, acetic acid, while *Sphingomonas* was the opposite. *Nitrospira* was positively correlated with N6-(1,2-dicarboxyethyl)-AMP, while negatively correlated with L-aspartic acid, 2-hydroxyphenylacetic acid, phenylacetaldehyde, and homogentisic acid. *Coccophora-langsdorfii* was negatively correlated with N6-(1,2-dicarboxyethyl)-AMP, while positively correlated with L-aspartic acid, 2-hydroxyphenylacetic acid, phenylacetaldehyde, and homogentisic acid.

In group CC7, metabolic pathways of phenylpropanoid biosynthesis, protein digestion and absorption, biosynthesis of various plant secondary metabolites, biosynthesis of siderophore group non-ribosomal peptides, styrene degradation, and glycolysis/gluconeogenesis were the key processes accounting for 23 DEMs of importance (e.g., benzene and substituted derivatives, phenylpropionic acid, phenols, carboxylic acid and derivatives, hydroxy acid and derivatives, cinnamic acid and derivatives, coumarin and derivatives [vanillin, fraxetin, ferulic acid, sinapic acid, salicylic acid, 4-hydroxybenzoic acid, etc.]). They were significantly (*p* ≤ 0.05) or extremely significantly (*p* ≤ 0.01) correlated with 12 bacterial genera. For example, *uncultured-bacterium-f-JG30-KF-CM45* was positively correlated with salicylic acid, and 5-hydroxyferulate, and *uncultured-bacterium-c-Acidimicrobiia* was negatively correlated with vanillin, (2S,3S)-2,3-dihydro-2,3-dihydroxybenzoate, 4-hydroxycinnamic acid, and 3-(2-hydroxyphenyl) propionic acid. *Sphingomonas* was negatively correlated with (2S,3S)-2,3-dihydro-2,3-dihydroxybenzoate, 3-(2-hydroxyphenyl) propionic acid. *Lysobacter* was negatively correlated with salicylic acid, 5-hydroxyferulate, and 4-hydroxybenzoic acid; *uncultured-bacterium-f-Gemmatimonadaceae* was positively correlated with ferulic acid and salicylic acid; *uncultured-bacterium-f-Xanthomonadaceae* was negatively correlated with sinapic acid, fraxetin, ferulic acid, salicylic acid, and 5-hydroxyferulate.

#### 3.6.2 The correlations between rhizosphere fungi and metabolites

Same as for rhizosphere bacteria, analyses were conducted to reveal the connections between key metabolic pathways producing DEMs and rhizosphere fungal communities for the different CC groups, as shown in Figure 7B. In CC1, 12 DEMs were significantly (*p* ≤ 0.05) or extremely significantly (*p* < 0.01) correlated with 7 fungal genera. Among them, *Chaetomium* was negatively correlated with (R)-mevalonic acid, inosinic acid, hydroquinone, and 4-hydroxyphenylacetaldehyde and positively correlated with 9(S)-HpOTrE, stearidonic acid, 9(S)-HOTrE, and alpha-linolenic acid. *Chalara* was positively correlated with (R)-mevalonic acid and inosinic acid and negatively correlated with 3,4-dihydroxybenzoic acid. *Cephaliophora* was negatively correlated with 4-hydroxyphenylacetaldehyde, (R)-mevalonic acid, and inosinic acid and positively correlated with traumatic acid, linoleic acid, and 3,4-dihydroxybenzoic acid. *Botryotrichum* was negatively correlated with (R)-mevalonic acid, hydroquinone, and inosinic acid and was positively correlated with traumatic acid, linoleic acid, 9(S)-HOTrE, 3,4-dihydroxybenzoic acid, and alpha-linolenic acid. *Entoloma* was positively correlated with traumatic acid, linoleic acid. *Rhizophagus* was positively correlated with traumatic acid, stearidonic acid, linoleic acid.

In group CC4, 13 DEMs were shown to be significantly (*p* ≤ 0.05) or extremely significantly (*p* ≤ 0.01) correlated with 7 genera. Among them, *Botryotrichum* and *Cephaliophora* were negatively correlated with (2S,3S)-2,3-dihydro-2,3-dihydroxybenzoate, 2-hydroxyphenylacetic acid, homogentisic acid, L-aspartic acid, and phenylacetaldehyde and positively correlated with acetic acid, d-lactic acid, and N6-(1,2-dicarboxyethyl)-AMP. *Cladosporium w*as positively correlated with L-aspartic acid and negatively correlated with acetic acid. *Kazachstania* and *Byssochlamys* were positively correlated with salicylic acid, L-aspartic acid, and 2-hydroxyphenylacetic acid and negatively correlated with gamma-aminobutyric acid, N6-(1,2-Dicarboxyethyl)-AMP, acetic acid. *Botryotrichum* and *Cephaliophora* were negatively correlated with (2S,3S)-2,3-dihydro-2,3-dihydroxybenzoate, L-aspartic acid, 2-hydroxyphenylacetic acid, phenylacetaldehyde, and homogentisic acid and positively correlated with D-lactic acid, acetic acid, and N6-(1,2-dicarboxyethyl)-AMP. *Wickerhamomyces* was positively correlated with salicylic acid, 2-hydroxyphenylacetic acid, and 2-isopropylmalic acid and negatively correlated with gamma-aminobutyric acid and N6-(1,2-dicarboxyethyl)-AMP. *Penicillium* was positively correlated with L-aspartic acid and phenylacetaldehyde and negatively correlated with acetic acid.

In group CC7, 23 DEMs were shown to be significantly (*p* ≤ 0.05) or extremely significantly (*p* ≤ 0.01) correlated with 11 genera in group D. *Cladosporium* was positively correlated with fraxetin, ferulic acid, sinapic acid, L-aspartic acid, 4-hydroxycinnamic acid, and isochorismate and negatively correlated with beta-alanine and acetic acid. *Chalara* was positively correlated with (2S,3S)-2,3-dihydro-2,3-dihydroxybenzoate, ferulic acid, 4-hydroxybenzoic acid, L-aspartic acid, phenylacetaldehyde, and isochorismate. *Kazachstania* and *Byssochlamys* were positively correlated with vanillin, fraxetin, ferulic acid, L-leucine, 5-hydroxyferulate, sinapic acid, psoralen, L-aspartic acid, 2-hydroxyphenylacetic acid, 4-hydroxycinnamic acid, and isochorismate. *Cephaliophora* was negatively correlated with (2S,3S)-2,3-dihydro-2,3-dihydroxybenzoate, fraxetin, ferulic acid, L-leucine, sinapic acid, psoralen, L-aspartic acid, 2-hydroxyphenylacetic acid, 4-hydroxycinnamic acid, isochorismate, and homogentisic acid. *Plectosphaerella* was positively correlated with ferulic acid, salicylic acid, and phenylacetaldehyde and negatively correlated with acetic acid. *Botryotrichum* was negatively correlated with (2S,3S)-2,3-dihydro-2,3-dihydroxybenzoate, ferulic acid, 4-hydroxybenzoic acid, L-aspartic acid, phenylacetaldehyde, and isochorismate. *Penicillium* was positively correlated with vanillin, (2S,3S)-2,3-dihydro-2,3-dihydroxybenzoate, fraxetin, ferulic acid, sinapic acid, L-aspartic acid, 4-hydroxycinnamic acid, and isochorismate and negatively correlated with beta-alanine and acetic acid. *Wickerhamomyces* was positively correlated with vanillin, fraxetin, L-leucine, salicylic acid, 5-hydroxyferulate, sinapic acid, 4-hydroxybenzoic acid, psoralen, and 2-hydroxyphenylacetic acid and negatively correlated with beta-alanine and L-methionine. *Entoloma* was negatively correlated with vanillin, (2S,3S)-2,3-dihydro-2,3-dihydroxybenzoate, L-aspartic acid, 4-hydroxycinnamic acid, isochorismate, homogentisic acid, and 3-(2-hydroxyphenyl) propionic acid.

### 3.7 The relationships between DEMs and microbiota in the spatial dimension

**Figure 8:**
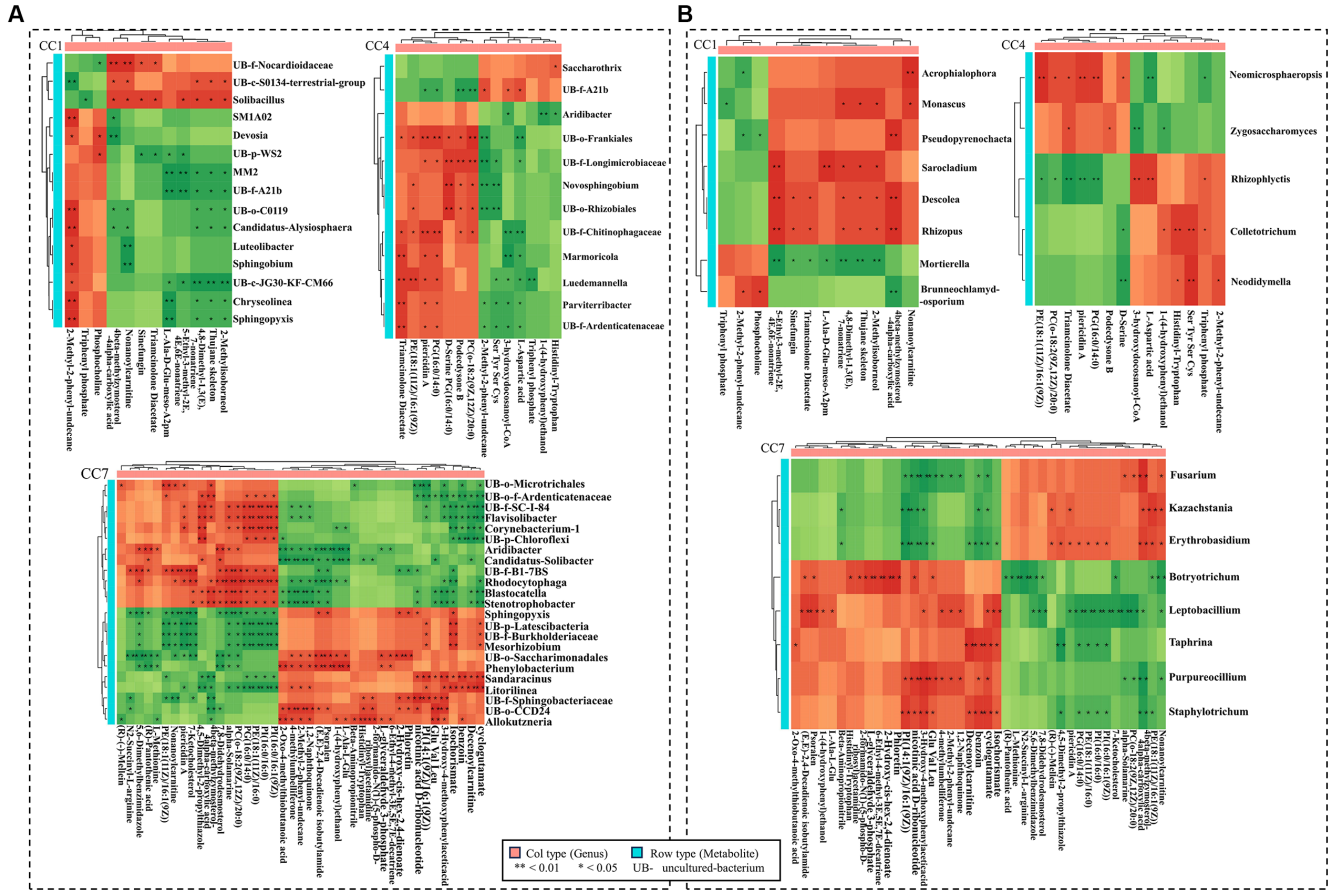
Correlation heatmap between GDEMs and the microbiota for rhizosphere and bulk soil. **(A)** The correlation heatmap between bacteria and the greatly differentially expressed metabolites (GDEMs). **(B)** The correlation heatmap between fungi and GDEMs. Every line is the significantly differentiated genus from rhizosphere and bulk soil with *t*-test (*p* < 0.05). Each column is GDEMs, green indicates negative correlation, red indicates positive correlation, ^*^indicates *p* < 0.05, and ^**^indicates *p* < 0.01.

Figure 8A shows the correlation between GDEMs and the bacteria in different CC groups (CC1–CC7), which were significantly different between rhizosphere (in vicinity of roots) and bulk soil (further away from roots; *p* ≤ 0.05). It should be noted that the bacterial genera shown were the ones that were significantly different between rhizosphere (in vicinity of roots) and bulk soil (further away from roots; *p* ≤ 0.05). *Sphingobium* was negatively correlated with nonanoylcarnitine, 3-(3-methylbutylidene)-1(3 h)-isobenzofuranone and positively correlated with 2-methyl-2-phenyl-undecane. *Uncultured-bacterium-f-Longimicrobiaceae* was negatively correlated with 2-methyl-2-phenyl-undecane, serine, tyrosine, serine, and cysteine (Ser, Tyr, Ser, Cys) and positively correlated with podecdysone B, (2R)-3-(icosanoyloxy)-2-[(9Z,12Z)-octadeca-9, and 12-dienoyloxy] propyl 2-(trimethylammonio) ethyl phosphate (pc (o-18: 2(9Z,12Z)/20:0)); *Stenotrophobacter* and *Blastocatella* were negatively correlated with 3-hydroxy-4-methoxyphenylacetic acid and 2-methyl-2-phenyl-undecane and positively correlated with alpha-solamarine, pc (o-18:2(9Z,12Z)/20:0) and 4beta-methylzymosterol-4alpha-carboxylic acid; *Flavisolibacter* and *Corynebacterium-1* were negatively correlated with decenoylcarnitine, while *Litorilinea* was positively correlated with decenoylcarnitine.

Figure 8B shows the correlation between the correlation between GDEMs and the fungal genera in different CC groups (CC1–CC7), which were significantly different between rhizosphere (in vicinity of roots) and bulk soil (further away from roots; *p* ≤ 0.05). *Mortierella* was negatively correlated with 4,8-dimethyl-1,3(e),7-nonatriene, thujane skeleton, 2-methylisoborneol, 5-ethyl-3-methyl-2e,4e,6e-nonatriene, L-Ala-D-Glu-Meso-A2pm, and triamcinolone diacetate; *Brunneochlamydosporium* was negatively correlated with 4beta-methylzymosterol-4alpha-carboxylic acid and positively correlated with phosphocholine; *Monascus* was positively correlated with 3-(3-methylbutylidene)-1(3 h)-isobenzofuranone; *Sarocladium* was positively correlated with 5-ethyl-3-methyl-2e,4e,6e-nonatriene and L-Ala-D-Glu-Meso-A2pm; *Zygosaccharomyce*s was negatively correlated with 3-hydroxydocosanoyl-CoA and positively correlated with triamcinolone diacetate, podecdysone B; *Botryotrichum* was negatively correlated with nonanoylcarnitine and positively correlated with 2-hydroxy-cis-hex-2,4-dienoate; *Fusarium* was positively correlated with 4beta-methylzymosterol-4alpha-carboxylic acid and negatively correlated with 3-hydroxy-4-methoxyphenylacetic acid, nicotinic acid d-ribonucleotide, glutamic acid, valine, and leucine (Glu, Val, Leu); *Kazachstania* was negatively correlated with 1-(9Z-tetradecenoyl)-2-(9Z-hexadecenoyl)-glycero-3-phospho-(1′-myo-inositol) (PI(14:1(9Z)/16:1(9Z))) and nicotinic acid d-ribonucleotide. In general, these results suggested that the root exudates of potato caused chemotaxis in these genera of microorganisms in soil, and their distribution in soil as a function of spatial dimension (close or away from roots) is an indication of their responses to chemical stimuli from the roots.

## 4 Discussion

Continuous cropping obstacles (CCOs) widely exist in crops, and the mechanisms are very complex. More and more studies indicated that autotoxins secreted by roots combine with rhizosphere microbial imbalance induced by root exudates were the main reasons of CCOs (Jin et al., 2019). Autotoxin secretion is controlled by plant metabolism, and microbiota changes in rhizosphere and bulk soil are responses to it. Both factors were investigated in this study for potato CCOs. It is reasonable to believe that the analytical methods developed in this study would be equally effective for assessment of CCOs for crops other than potato.

### 4.1 Metabolic pathways in potato controlling root exudates affected by continuous cropping

Root exudates often change when plants are under duress and responding to environmental stressors. The resulted autotoxicity of root exudates was regarded as one of the main reasons of CCOs. As in the cases of *Angelica sinensis*, *Lilium davidii* var. *unicolor*, *Panax quinquefolium*, *Nicotiana tabacum* L., and *P. notoginseng*, the increase of autotoxic allelochemicals in root exudates due to CC was identified as a key reason for failure of replanting (He et al., 2009; Wu Z. J. et al., 2015; Yang et al., 2015; Deng et al., 2017; Xin et al., 2019). In this study, it was discovered that potato root exudates were significantly changed as a function of CC years. Compared with rotation control (CC0), the changes of root exudates of potato from group CC1 could be attributed to α-linolenic acid metabolism, benzoate degradation, biosynthesis of plant secondary metabolites, and biosynthesis of plant hormones. Especially, α-linolenic acid synthesis was downregulated. Linolenic acid was the precursor of jasmonic acid (JA), and JA is a lipogenic plant hormone that regulates the defensive responses of plants to biological and abiotic stresses (Zhao et al., 2014; Balfagon et al., 2019; Zhu et al., 2021; Pyo et al., 2022). Downregulated linolenic acid may suggest that stress levels in potato plants of group CC1 were not increased very much compared to that of group CC0. Meanwhile, for groups CC4 and CC7, metabolic pathways of styrene degradation and biosynthesis of siderophore group non-ribosomal peptides were significantly altered, and the production of phenolic acids (e.g., homogentisic acid and salicylic acid) went up significantly. Homogentisic acid is the precursor for the biosynthesis of α-tocopherol, which regulates the concentration of reactive oxygen and plant hormones in response to stress (Munné-Bosch and Alegre, 2002). Its upregulation was in sharp contrast to the downregulation of linolenic acid-JA synthesis in plants of group CC1. The effects of salicylic acid were concentration-dependent; when the concentration exceeded 0.5 mM, it was shown to significantly affect the mineral absorption of plants (Harper and Balke, 1981), stomatal movement, and chlorophyll content (Manthe et al., 1992; Pancheva et al., 1996). It was also shown to reduce protein content and photosynthetic rate in barley plants (Pancheva et al., 1996). In the range of 3–5 mM, it was shown to completely inhibit the germination of maize embryo (Guan and Scandalios, 1995). In group CC7, metabolic pathway of phenylpropanoid biosynthesis and biosynthesis of various plant secondary metabolites were also significantly changed, and the production of phenolic acids (e.g., sinapic acid, ferulic acid, 4-hydroxycinnamic acid, 4-hydroxybenzoic acid, and vanillin) and coumarin (e.g., psoralen and fraxetin) went up. Vanillin was shown to be connected to changes in microbial communities of the cucumber rhizosphere and the replant failure of eggplant due to CCOs (Chen et al., 2011; Jia et al., 2018; Zhang et al., 2018). Oxidative stress arising from elevated ferulic acid was connected to cellular dysfunction and cell death, and inhibition of the growth of the seedlings (Pergo and Ishii-Iwamoto, 2011; Mandavikia et al., 2017). 4-Hydroxybenzoic acid was shown to regulate grapevine secretion and could cause replant disease (Wang et al., 2019). Psoralen and fraxetin are coumarins, whose effects on plant growth were also dosage-dependent. When the concentration of coumarin was at ~680 μM, the root growth of cucumber and maize seedlings was completely inhibited (Pergo et al., 2008). 4-Hydroxycinnamic acid (p-coumaric acid) could also significantly inhibit crop growth, such as cucumber (Zhou and Wu, 2012), strawberry (Chen et al., 2020), and asparagus (Kato-Noguchi et al., 2017). The elevation of phenolic acids and coumarin production in groups CC7 clearly was one of the main reasons of yield drop in this group. These observations also confirmed that none of these autotoxic allelochemicals were unique to potato, they belong to a group of compounds that could affect various plants, and monitoring their levels could hold keys to understand CCOs in various crops.

In addition to the DEMs involved in the above metabolic pathways, other DEMs increased in a CC-year dependent pattern (Supplementary Table S8), including potential autotoxins: phenols (1-(4-hydroxyphenyl) ethanol, homogentisic acid, 2-aminophenol, purpurogallin), alkaloids (conhydrine), flavonoids (phloretin), coumarins (psoralen), as well as 5-phenyl-4-pentenyl-hydroperoxide (PPHP), 2,5-diketo-d-gluconate, 5-methylaminomethyl-2-thiouridine, N-acetyl-D-proline, benzoin, urodiolenone, and benzyl butyl phthalate. Among them, 1-(4-hydroxyphenyl) ethanol was increased in group CC4, while 1,2-naphthoquinone, 4-methylumbelliferone, 1-(4-hydroxyphenyl) ethanol, phloretin, psoralen, and beta-aminopropionitrile were all significantly increased in group CC7. Studies have found that in addition to coumarins (Yan et al., 2016), phenols (Chaki et al., 2020) and alkaloids (Lei et al., 2021) also indicate that the plants were under oxidative stress. PPHP is a hydroperoxide, an initial product of lipid peroxidation (Weller et al., 1985), and lipid peroxidation is a major indicator of oxidative damage in plants (Chen et al., 2021; Fardus et al., 2021). 2,5-Diketo-d-gluconate is a key intermediate in the production of l-ascorbic acid (vitamin C; Son et al., 2022), which is a main antioxidant in plants and plays important roles in alleviating excessive activities of oxidative free radicals caused by many abiotic stresses (Gallie, 2013). Exogenous application of 4-methylumbelliferone to *Arabidopsis thaliana* seeds before seedling formation could affect seed germination, resulting in reduced primary root growth, root hair formation, irregular root cap shedding, and reorganization of actin cytoskeleton in root tip (Li et al., 2011). Phloretin also significantly inhibited the growth of primary roots, lateral roots, and leaves of *Arabidopsis thaliana* (Smailagic et al., 2022). The changes in these DEMs related to plant stress responses all indicated the effects of CC on the potato plants. As a general trend, the more CC years, the more autotoxin production in potato plants.

### 4.2 Microflora changes affected by continuous cropping

Changes in microflora and its imbalance were also important contributing factors to CCOs (Tan et al., 2017; Shen et al., 2018; Li X. G. et al., 2020). Studies found that harmful fungi increased and beneficial bacteria and actinomycetes decreased in soil of potato field undergoing contentious cropping (Qin et al., 2017a; Zhao et al., 2020); consequently, the microbial community structure in soil became unbalanced, with overgrowth of harmful microorganisms such as *Fusaria*, and inhibited plant root growth (Qin et al., 2017a). This study also found that beneficial bacteria (*Sphingomonas*, *Lysobacter*, *Altererythrobacter*, *Rhizophagus*, *Septoglomus*, *Mortierella,* and *Funneliformis*) all were significantly reduced, and harmful fungi (*Cladosporium*, *Plectosphaerella*, *Zasmidium*, *Aspergillus*, and *Chalara*), on the other hand, were significantly increased under CC, especially in groups CC4 and CC7. Among them, *Sphingomonas* was plant growth-promoting endophytic bacteria (PGPEB; Khan et al., 2017), which plays a role in promoting plant growth (Khan et al., 2014; Pan et al., 2016). *Lysobacter* spp. were reported to reduce diseases caused by plant pathogens in *Cucumis sativus* Linn (Folman et al., 2004; Postma et al., 2008), *Oryza sativa* (Ji et al., 2008), *Piper nigrum* Linn (Ko et al., 2009), *Vitis vinifera* (Puopolo et al., 2014), *Spinacia oleracea* L. (Islam et al., 2005), and *Lycopersicon esculentum* (Puopolo et al., 2010). *Altererythrobacter* is genera of bacteria involved in C cycling (An et al., 2022) that could bring ecological benefits (Liu et al., 2022). *Septoglomus*, *Funneliformis,* and *Rhizophagus* (Rodriguez-Caballero et al., 2017; Cui et al., 2018; Todeschini et al., 2018) could colonize on the roots of most terrestrial plant species and improve plant growth, nutrient uptake, and biotic/abiotic stress resistance and tolerance. *Mortierella alpina* was reported to help *Panax ginseng* resist *Fusarium oxysporum* infection by regulating the fungal community in the root (Wang Y. et al., 2022), and *Mortierella capitata* was reported to promote crop growth directly by altering gene expression levels in the plant roots and indirectly via interacting with indigenous rhizosphere bacteria (Li F. et al., 2020). *Cladosporium* (Virginia et al., 2021; Wang T. et al., 2022), *Plectosphaerella* (Garibaldi et al., 2010; Xu et al., 2014), *Zasmidium* (Laranjeira et al., 2020), *Aspergillus* (Ali et al., 2022), and *Chalara* (Wang et al., 2021) were all plant pathogens, which mainly presented in rhizosphere soil and significantly increased with CC years as shown in this study.

### 4.3 Correlation between root exudates and microflora

Root exudates as substrates and/or signal molecules for microbe are the main driver of rhizosphere microflora (Zhalnina et al., 2018). Plant–microbe interactions mediated by root exudates could facilitate plant CCO and resulted in plant diseases (Wu et al., 2023). For instance, ginseng roots exudates could trigger bloom of the ginseng soft-rot bacteria, which is the culprit for one of major bacterial diseases that affect ginseng plants and the cause for drop in both the yield and quality of ginseng roots (Lei et al., 2017). In tobacco root exudates, cinnamic, myristic, and fumaric acids were identified as attractants to induce the colonization and infection of the roots by *Ralstonia solanacearum*, which led to one of the most serious soil-borne diseases in tobacco cultivation (Li et al., 2017). Previous study found that the root exudates of *Rehmannia glutinosa* could inhibit the growth of *Pseudomonas* sp. W12, a beneficial bacterium and promote the growth and toxin production of pathogenic *Fusarium oxysporum* (Wu L. K. et al., 2015). In the soil of monocropping field of *Radix pseudostellariae*, vanillin (e.g., phenols) was shown to promote the colonization and growth of *Kosakonia sacchari*, the pathogen of *R. pseudostellariae*, and increased the probability of disease occurrence (Wu et al., 2017). In this study, significant (*p* ≤ 0.05) or extremely significant (*p* ≤ 0.01) positive correlations were established for potato crops between pathogenic microbes and root exudates; *Cladosporium* was positively correlated with ferulic acid (phenolic acids; *p* ≤ 0.01), sinapic acid (phenolic acids; *p* ≤ 0.01), L-aspartic acid (amino acid; *p* ≤ 0.05), 4-hydroxycinnamic acid (phenolic acids; *p* ≤ 0.05), fraxetin (coumarin; *p* ≤ 0.05), and isochorismate (*p* ≤ 0.05), *Chalara* was positively correlated with ferulic acid (*p* ≤ 0.01), 4-hydroxybenzoic acid (phenolic acids; *p* ≤ 0.05), L-aspartic acid (*p* ≤ 0.01), and isochorismate (*p* ≤ 0.01), and *Plectosphaerella* was positively correlated with ferulic acid (*p* ≤ 0.01) and salicylic acid (*p* ≤ 0.05). In contrast, the opposite was true for beneficial bacteria; *Sphingomonas* was negatively correlated with 2-hydroxyphenylacetic acid (phenolic acids; *p* ≤ 0.05), phenylacetaldehyde (*p* ≤ 0.01), and homogentisic acid (phenolic acids; *p* ≤ 0.05), and *Nitrospira* was negatively correlated with 2-hydroxyphenylacetic acid (*p* ≤ 0.05), phenylacetaldehyde (*p* ≤ 0.05), and homogentisic acid (*p* ≤ 0.05). *Lysobacter* was negatively correlated with salicylic acid (*p* ≤ 0.01), 5-hydroxyferulate (*p* ≤ 0.01), and 4-hydroxybenzoic acid (*p* ≤ 0.05), and *Sphingomonas* was negatively correlated with 2-phenylacetaldehyde (*p* ≤ 0.01).

Closer investigation revealed some complex patterns. For example, the relative abundance of *Fusarium* in the rhizosphere of CC crops, surprisingly, did not peak in group CC7 but in group CC4. Furthermore, *Fusarium* were significantly enriched in the bulk soil of group CC7. This “puzzle” could be solved from the spatial correlations between DEMs and microbiota. Our study showed that the increase of 4-methylumbelliferon (from bulk to rhizosphere) was negatively correlated with the changes in relative abundance of *Fusarium* in the bulk soil, which could be attributed to coumarins. Studies have shown that coumarin could have a strong inhibitory effect on *Fusarium* in a dosage-dependent way. At high concentrations, they could inhibit mycelial growth, sporulation, and pathogenicity-related enzyme activities (Wu et al., 2008). Hence, the accumulation of high coumarins in the rhizosphere of group CC7 could inhibit the growth of *Fusarium*. However, *Fusarium* was still highly enriched in the bulk soil, which attributed to the much-reduced yield of potato from the group CC7. Finally, stimulation of psoralen accumulation by biotic elicitors such as yeast extract and chitosan has previously been observed in the cell cultures of plant species, *viz.*, *Calendula officinalis* (Wu et al., 2008), *Sorbus aucuparia* (Gaid et al., 2011), and *Abrus precatorius* (Karwasara and Dixit, 2009; Karwasara et al., 2011). In this study, psoralen was shown to be positively correlated with *Kazachstania* (*p* ≤ 0.05), *Byssochlamys* (*p* ≤ 0.05), and *Wickerhamomyces* (*p* ≤ 0.01), and these fungi were reported to promote plant growth by producing plant hormones and other growth regulators (Nassar et al., 2005; Cloete et al., 2009; Xin et al., 2009; Amprayn et al., 2012; Nutaratat et al., 2014) or interacted indirectly with symbiotic microorganisms such as arbuscular mycorrhizal fungi (AMF; Fracchia et al., 2003; Boby et al., 2008). In general, the knowledge gained on relationships between metabolites and microbial communities could provide guidance for the regulation/manipulation of rhizosphere microflora via biotechnology (e.g., microbial fertilizer) to overcome continuous cropping obstacles and improve both yield and quality of potato crops.

## 5 Conclusion

The results showed continuous cropping of potato changed the metabolic pathways in potato that significantly changed alpha-linolenic acid metabolism in plants from 1 year CC field, styrene degradation, biosynthesis of siderophore group non-ribosomal peptides, phenylpropanoid biosynthesis, and biosynthesis of various plant secondary metabolites in plants from 4 to 7 years CC field, and increased phenols, flavonoids, coumarins, and alkaloids in root exudates. Continuous cropping of potato beyond 4 years changed their metabolism as reflected in the plant root exudates to drive rhizosphere microflora toward the direction of reducing beneficial bacteria and promoting harmful fungi, which need to be better controlled to reduce the impact of CCO on potato production.

## Data availability statement

The datasets presented in this study can be found in online repositories. The names of the repository/repositories and accession number(s) can be found in the article/Supplementary material.

## Author contributions

YX: Data curation, Formal analysis, Funding acquisition, Investigation, Writing – original draft. PZ: Funding acquisition, Methodology, Project administration, Writing – original draft. WZ: Conceptualization, Funding acquisition, Investigation, Methodology, Project administration, Supervision, Writing – original draft, Writing – review & editing. CY: Formal analysis, Methodology, Writing – review & editing. ZL: Conceptualization, Supervision, Writing – review & editing.
