# Supplementary figures and images for: Continuous cropping of potato changed the metabolic pathway of root exudates to drive rhizosphere microflora
Source: Front Microbiol. 2024 Jan 5;14:1318586. doi: 10.3389/fmicb.2023.1318586 (PMC10797025; doi:10.3389/fmicb.2023.1318586)

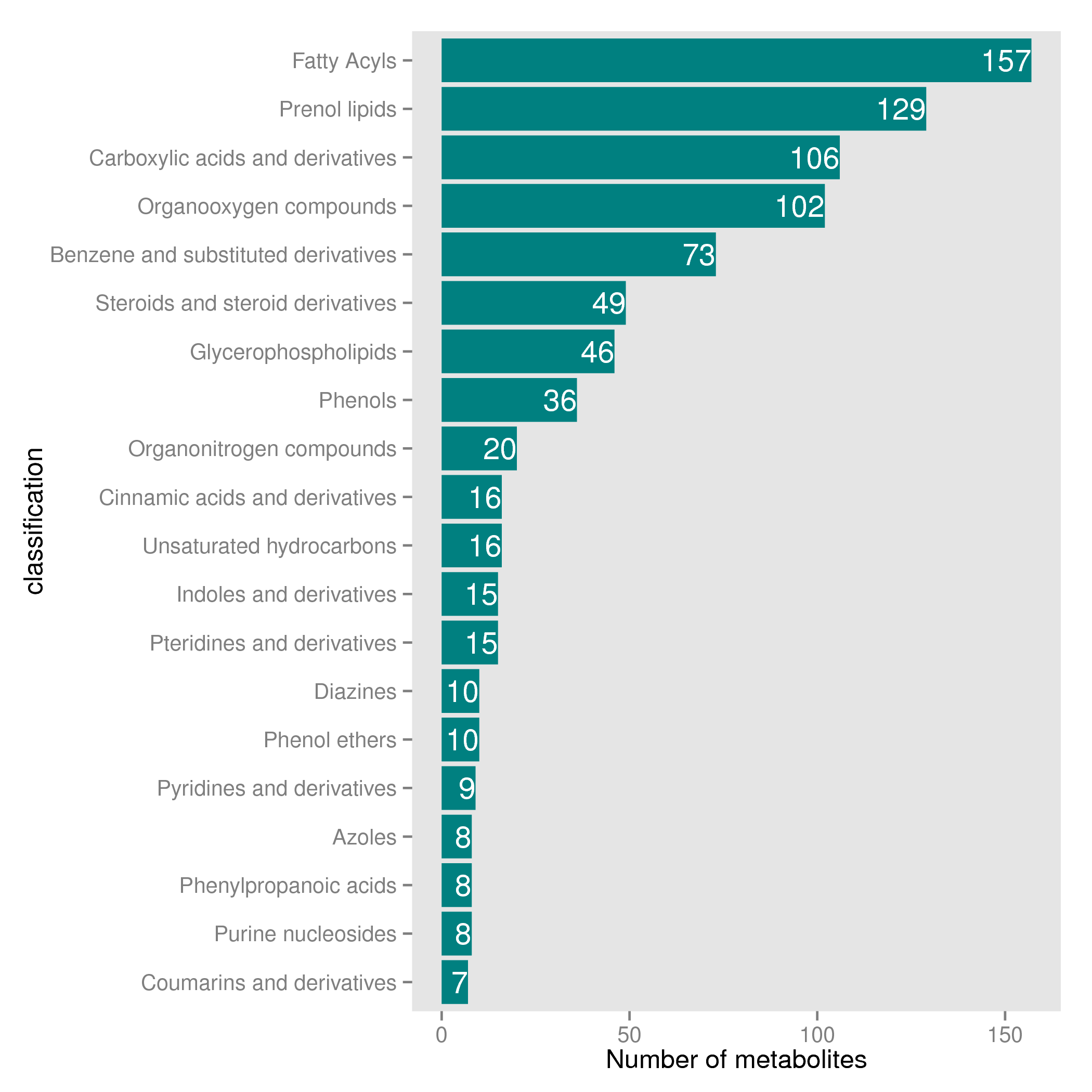

Supplement: Supplementary file 1 [file Data_Sheet_1.zip › Supplementary Fig1.png]
